# Study on the Energy Absorption Performance of Triply Periodic Minimal Surface (TPMS) Structures at Different Load-Bearing Angles

**DOI:** 10.3390/biomimetics9070392

**Published:** 2024-06-27

**Authors:** Yongtao Lyu, Tingxiang Gong, Tao He, Hao Wang, Michael Zhuravkov, Yang Xia

**Affiliations:** 1 Department of Engineering Mechanics, Dalian University of Technology, Dalian 116024, China; 2 DUT-BSU Joint Institute, Dalian University of Technology, Dalian 116024, China; 3 Wuhan Second Ship Design and Research Institute, Wuhan 430205, China; 4 Faculty of Mechanics and Mathematics, Belarusian State University, 220030 Minsk, Belarus

**Keywords:** triply periodic minimal surface, additive manufacturing, quasi-static compression, energy absorption, different load-bearing angles

## Abstract

As engineering demands for structural energy absorption intensify, triply periodic minimal surface (TPMS) structures, known for their light weight and exceptional energy absorption, are increasingly valued in aerospace, automotive, and shipping engineering. In this study, the energy absorption performance of three typical TPMS structures was evaluated (i.e., Gyroid, Diamond, and IWP) using quasi-static compression tests at various load-bearing angles. The results showed that while there is little influence of load-bearing angles on the energy absorption performance of Gyroid structures, its energy absorption is the least of the three structures. In contrast, Diamond structures have notable fluctuation in energy absorption at certain angles. Moreover, IWP (I-graph and Wrapped Package-graph) structures, though highly angle-sensitive, achieve the highest energy absorption. Further analysis of deformation behaviors revealed that structures dominated by bending deformation are stable under multi-directional loads but less efficient in energy absorption. Conversely, structures exhibiting mainly tensile deformation, despite their load direction sensitivity, perform best in energy absorption. By integrating bending and tensile deformations, energy absorption was enhanced through a multi-stage platform response. The data and conclusions revealed in the present study can provide valuable insights for future applications of TPMS structures.

## 1. Introduction

As technology continues to advance, biomimetics, as an interdisciplinary field that imitates natural biological structures and functions, is increasingly gaining attention. The development of biomimetic structures transforms biological structures from nature into inspirations for engineering design, leading to the creation of more efficient and sustainable technological innovations. By drawing from nature’s designs, humans can explore and harness solutions that have been optimized through long evolutionary processes in nature, bringing about new breakthroughs and advancements in various fields. Biomimetic structures pave the way for technological advancement while also sparking exploration and research into the biological field.

Inspired by nature, TPMS structures have been discovered, and their advantage lies in the fact that the curvature in all three orthogonal directions is zero [1,2,3]. Compared to lattice structures such as octet, cubic, body-centered cubic (BCC), or face-centered cubic (FCC), the advantage of reducing the stress concentration effects are exhibited in TPMS structures, which can enhance their mechanical performance, such as energy absorption capacity [4,5,6,7]. In the field of energy absorption in engineering practice, TPMS structures have many application examples and prospects, for example, in vehicle bumpers, sports protective gear, architectural structures, industrial equipment, sports shoe insoles, and so on [8].

The absorption of external impact and collision energy in TPMS structures is achieved primarily through internal damage and deformation, with compression deformation playing a crucial role in the process of structural energy absorption. Consequently, scholars have placed significant emphasis on studying the compressive behavior of TPMS structures [9,10,11,12,13,14,15,16,17]. Lu et al. [18] compared the compressive performances of the Gyroid and Primitive structures, finding that greater deformation tolerance was demonstrated in compression tests in the Gyroid structure. Zhang et al. [19] conducted compression tests on shell-based TPMS structures and observed more uniform stress distribution in Gyroid and Diamond structures, whereas the localized buckling of the walls was exhibited in the Primitive structure. Furthermore, Maskery et al. [20] investigated the failure modes under compression, noting that the unit cell size plays a crucial role in determining failure mechanisms, with smaller unit sizes helping to prevent structural failure under low strain conditions. Interestingly, TPMS structures subjected to heat treatment were found to absorb more energy. By adjusting the thickness of the structural walls, gradient TPMS structures can be created to enhance performance beyond that of homogeneous structures. Yang et al. [21] studied the characteristics of energy absorption of gradient TPMS structures, revealing that the capacity for energy absorption decreases with increasing unit cell size. Compared to uniform structures, more energy was able to be absorbed in gradient Primitive structures [22]. In contrast, the capability of energy absorption of gradient Gyroid structures was similar to their uniform counterparts. Maskery et al. [23] explored the compressive deformation behaviors of Gyroid, Diamond, and Primitive structures, noting that a longer plastic stage contributes to higher efficiency of energy absorption. Additionally, compared to Primitive or Gyroid structures, higher strains can be withstood before failure in IWP and Neovius structures. Therefore, superior performance in energy absorption was demonstrated [17,24].

To further elucidate the superior performance of TPMS structures in energy absorption, Oraib et al. [25] analyzed and compared the mechanical properties of beam-based TPMS, shell-based TPMS, and classic lattice structures. The study revealed that shell-based TPMS structures exhibit behavior dominated by tensile deformation, while beam-based TPMS structures exhibit bending-dominated behavior. Moreover, Oraib et al. [26] found that among these structures, shell-based TPMS structures generally exhibited superior performance. Additionally, given that TPMS structures can also be considered composites of solid and air, interesting experiments were undertaken by replacing the air phase with other materials to create interpenetrating phase composites with TPMS, aiming to enhance mechanical performance [27,28,29,30,31,32]. Speirs et al. [33] investigated the fatigue behavior of TPMS scaffolds manufactured from NiTi shape memory alloys using Selective Laser Melting (SLM). Compared to octahedral units, fatigue resistance was enhanced due to smooth surfaces of TPMS structures. Recent studies have also analyzed the fracture toughness of TPMS [34,35]. Khan and colleagues examined the viscoelastic behavior of TPMS under both the time and frequency domains [36]. Their experimental results demonstrated that the IWP structure performed excellently under uniaxial loading, with the Primitive structure showing the highest shear and bulk responses. Furthermore, leveraging the advantages of TPMS, researchers have developed novel porous structures to achieve better performance [37]. Cao et al. designed Primitive lattices by generating lattice struts along smooth surfaces [38]. Compared to traditional lattices, this new structure can offer superior mechanical properties and enhance the ability of energy absorption. Additionally, Maskery and Ashcroft developed a new type of honeycomb based on the Gyroid surface by altering the cell wall shapes, which yielded novel deformation and post-yield hardening under in-plane loads [39].

As can be seen from the previous studies, research on the energy absorption of TPMS porous structures has become an international hotspot. However, there are still shortcomings in existing studies. In particular, there is currently a lack of systematic research on the energy absorption characteristics of TPMS structures in different load directions. This is crucial for assessing the stability and adaptability of structures when facing multi-directional loadings. If the energy absorption characteristics of TPMS structures under different angles of loadings can be analyzed in detail, and the changing patterns of their performance can be revealed, this will not only provide important guidance for the practical application of TPMS structures, but also lay a theoretical foundation for the future targeted optimization of TPMS structures in terms of energy absorption performance.

In this study, the TPMS structures were fabricated from 316L steel powder using SLM technology. Experimental evaluations of the compressive behavior, the overall deformation patterns, and energy absorption capabilities of Gyroid, Diamond, and IWP (I-graph and Wrapped Package-graph) structures under quasi-static loads at various load-bearing angles were conducted in this study. Furthermore, an analysis of the structural energy absorption changes induced by deformation patterns was performed, elucidating the relationship between the patterns of structural deformation and the capabilities of energy absorption.

## 2. Materials and Methods

### 2.1. Selection of Materials

In this study, 316L steel was selected as the preferred material due to its high strength and superior ductility. It is a common material used in energy-absorbing devices such as automotive energy-absorption boxes and crash beams [40]. The chemical composition of 316L steel is presented in Table 1. Additionally, 316L steel is favored for its manufacturability and cost effectiveness, making it widely applicable in various industrial fields. The mechanical properties of the 316L steel used in this study are shown in Table 2.

### 2.2. Design of TPMS Structures

In this study, the Gyroid, Diamond, and IWP structures were selected for energy absorption research, primarily due to their differences in anisotropy. Among them, the least obvious anisotropy was exhibited in the Gyroid structure, while the most obvious anisotropy was exhibited in the IWP structure [41]. Since anisotropy was utilized to describe the mechanical properties of the structure in the elastic phase, this study selected three structures with different anisotropies for research to explore their energy absorption performance after yielding.

**Figure 1:**
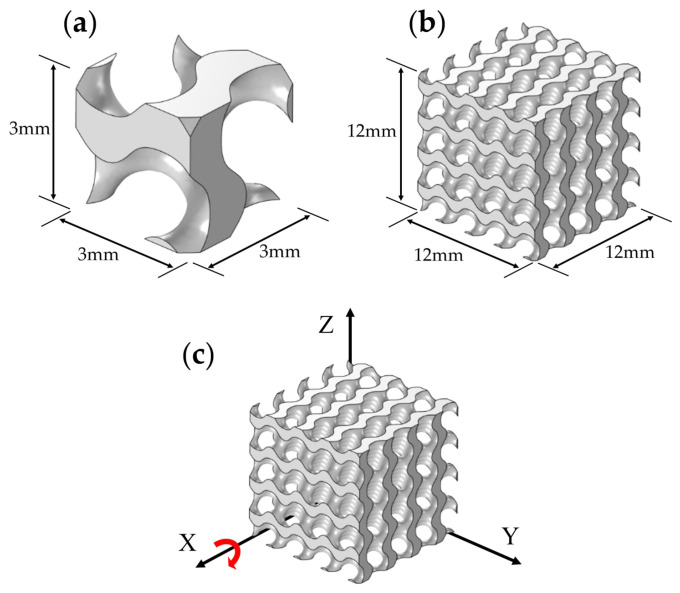
The model of the TPMS structures: (**a**) cell size; (**b**) the size of the 4 × 4 × 4 model; (**c**) schematic diagram of changing the load-bearing angle of the Gyroid structure along the X-axis. The red arrow indicates the position of the axis of rotation.

**Figure 2:**
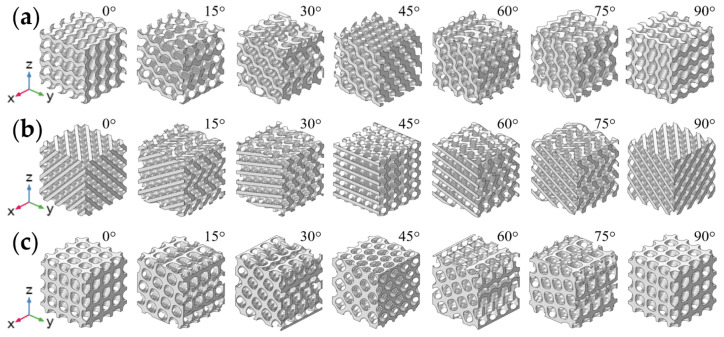
Three-dimensional models with different load-bearing angles: (**a**) Gyroid; (**b**) Diamond; (**c**) IWP. (Note: The axis of rotation was the X-axis. The coordinate axis in Figure 2 was consistent with Figure 1c, so the position of the axis of rotation can be referenced to Figure 1c).

In this study, three types of TPMS structures (i.e., Gyroid, Diamond, and IWP) were designed using the open-source TPMS generator Flatt pack (UoN, Nottingham, UK) [42]. The dimensions of the samples were 12 mm × 12 mm × 12 mm, with each unit cell sized at 3 mm × 3 mm × 3 mm, as shown in Figure 1. The three TPMS structures (Gyroid, Diamond, and IWP) were rotated along the X-axis at angles of 0°, 15°, 30°, 45°, 60°, 75°, and 90°, resulting in 21 different models, as shown in Figure 2. The TPMS models were converted into 3D stereolithography (STL) files and imported into Materialise Magics (Materialise Inc., Leuven, Flemish, Belgium) to add supports for subsequent 3D printing.

### 2.3. Fabrication of TPMS Samples

The fabrication of TPMS samples was conducted using the metal 3D printer Yongnian YLM-150 (Jiangsu Yongnian Inc., Kunshan, Jiangsu, China), with a manufacturing precision of 0.05 mm. The printing parameters for the machine are detailed in Table 3. Prior to printing, it is necessary to slice the STL format of the TPMS model to generate the appropriate layered data for printing. Once the model preparation is complete, the material reservoir of the printer must be loaded with metal powder specifically used for sample molding.

**Figure 3:**
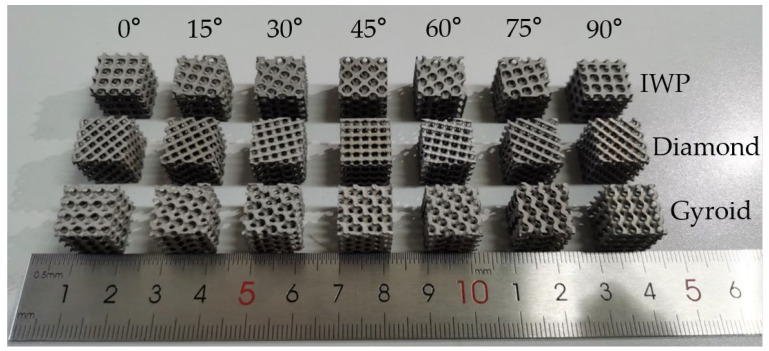
316L steel samples of multi-angle TPMS structures fabricated by SLM.

After the raw material is loaded, the model undergoes a melting and molding process. It is important to note that post-processing steps such as the removal of support structures and surface treatment are crucial after the extraction of the samples. These steps help to eliminate residual stresses within the model, thereby minimizing their potential impact on the mechanical properties of the experimental samples. The experimental samples produced by 3D printing are shown in Figure 3. To diminish potential errors during the experiment, six samples of each structure were printed for testing.

**Figure 4:**
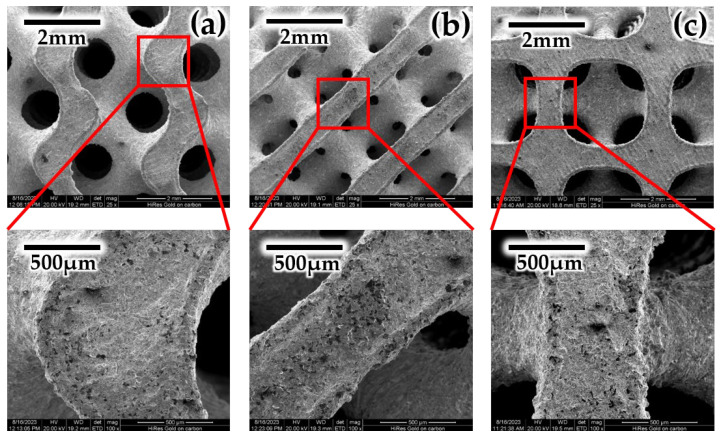
Surface topography of metal printed samples: (**a**) Gyroid, (**b**) Diamond, and (**c**) IWP.

To ensure the reliability and accuracy of subsequent experiments, a quality analysis is performed after the completion of sample fabrication. This process not only helps to confirm the integrity of the printed samples but also enables the precise assessment of their printing accuracy, serving as a crucial guarantee for the success of the experiments. Images captured by scanning electron microscopy (SEM) are shown in Figure 4. The microstructure of the surface of samples is displayed, revealing a slightly rough surface that broadly reflects the shape and detailed features of the original model. This slight roughness may be attributed to the natural texture formed during the bonding or cooling process of the steel powder layers during printing [25,26]. It is noteworthy that almost no grains formed by the melted steel powder are found on the sample surface, indicating the stability of the material and the high precision of the printing technology during the forming process. This stability is crucial for ensuring the structural consistency and functional reliability of the printed samples. Furthermore, by comparing the pore diameters of the original model and the printed sample, it is found that the difference between them is minimal and almost negligible. Moreover, the outstanding performance of SLM was demonstrated due to the high level of consistency in detailed fabrication. The quality analysis of the printed sample confirms that samples printed using SLM exhibit high printing accuracy and good quality, thus ensuring the performance of the experimental samples in subsequent experiments.

### 2.4. Quasi-Static Compression Test

**Figure 5:**
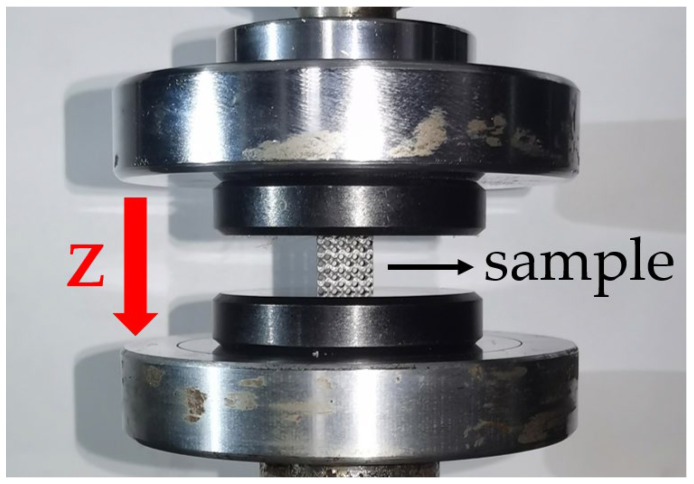
Quasi-static compression test setup. The red arrow indicates the Z direction of loading.

The ability of energy absorption of TPMS structures is fundamentally linked to its stress–strain relationship. In this study, the stress–strain relationships of TPMS structure samples were determined through quasi-static compression testing. In the experiments, a SANS compression testing machine was utilized, which can deliver a maximum load of 50 kN. A consistent quasi-static compression mode was applied at a rate of 0.5 mm/min. The setup of the compression tests conducted in this study is depicted in Figure 5. At the start of the experiment, this study ensured that the upper and lower compression platens were parallel and the sample was centered on the platform. On the compression testing machine, sensors were connected to the upper and lower platens. As compression proceeded, these sensors automatically collected reaction forces and displacement data from the platens. These data were processed by a computer and plotted into force–displacement curves.

**Figure 6:**
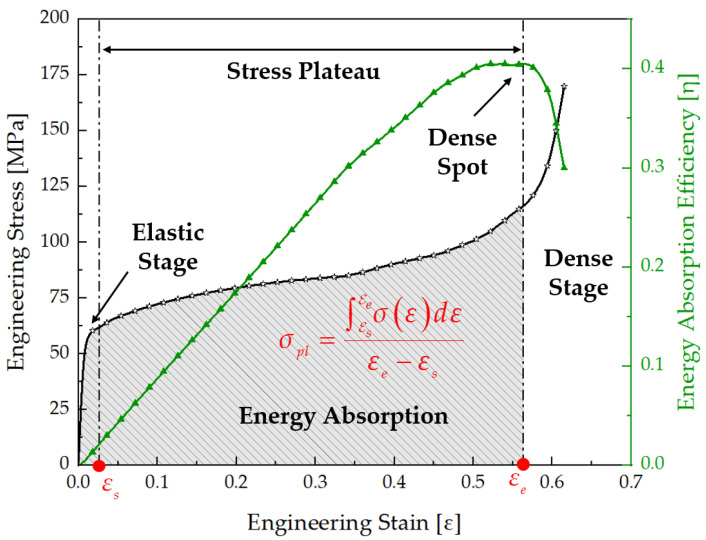
Schematic diagram of the energy absorption of structures.

In this study, the force–displacement curves were converted into the stress–strain curves for subsequent analysis. The engineering stress was determined by dividing the reaction force by the nominal cross-sectional area (144 mm^2^). The engineering strain was determined by dividing the displacement data of the upper platen collected by sensors by the initial dimension of the structure (12 mm). During the process of energy absorption failure of the TPMS structure under quasi-static compressive load, the stress–strain curves showed in three distinct stages, as shown in Figure 6. These three stages are the elastic stage, the stress plateau stage, and the densification stage [40].

In the elastic stage, the characteristics of elastic deformation were exhibited, and a linear elastic behavior was shown in the stress–strain relationship at this point. As energy absorption of the structure begins, its internal stress gradually increases, eventually leading to damage, at which point the plastic stage of the structure begins to emerge. At this stage, the stress variation of the structure exhibits nonlinear characteristics, while damage begins to occur internally within the structure. These damages may include elastic buckling, plastic yielding, or fracture, or a combination of these damage mechanisms. In this study, 316L steel was selected as the material of TPMS structures, known for its excellent ductility characteristics. Therefore, during structural collapse, significant fractures are typically not observed. The primary form of damage is a combination of crushing and plastic yielding. Additionally, in the plastic stage, the stress value of the structure often rises slowly or tends to stabilize, and hence, this stage is referred to as the stress plateau stage. At this stage, the internal stress of the structure is defined as the plateau stress (*σ_pl_*), which is the average level of stress during the stress plateau stage, starting from the yield stress and ending when the structure begins to densify, as described in Equation (1).

(1) $$\sigma_{pl}=\frac{\int_{E_{s}}^{E_{e}}\sigma \left(\varepsilon \right)d\varepsilon }{\varepsilon_{e}-\varepsilon_{s}}$$

where $\varepsilon_{s}$ denotes the initial strain at the onset of the platform stage, which also corresponds to the strain at which yielding occurs in the structure. At this point, the stress in the structure is equal to the yield strength of the material. $\varepsilon_{e}$ represents the strain at the end of the platform stage, also referred to as the densification strain, at which point the structural stress reaches the ultimate strength of the material. As the damage and deformation continue to occur, the densification stage of the structure begins to emerge progressively. During this stage, the internal stress within the structure begins to rise rapidly. The shape of the structure gradually becomes more compact and dense. As compression continues, the unique characteristics of the structure gradually diminish, exhibiting properties similar to its solid material [43].

The capacity for specific energy absorption is one of the crucial metrics for evaluating metamaterials. It quantifies the energy absorbed per unit mass of a structure, commonly referred to as the specific energy absorption (SEA). Structures with a higher SEA can meet the energy absorption requirements in engineering while saving solid materials. The SEA of the structure reflects its ability to absorb energy when subjected to external loads or impacts. The expression for SEA is shown in Equation (2).

(2) $$SEA=\frac{w_{v}}{M}$$

where SEA represents the specific energy absorption of the structure (kJ/kg); *M* denotes the mass of the structure per unit volume, with a unit of kg/m^3^, which can be calculated by multiplying the density of the structure by its total volume; and $w_{v}$ represents the total energy absorbed w by unit volume structure when $\varepsilon =\varepsilon_{e}$, with a unit of kJ/m^3^. The expression of w is presented in Equation (3).

(3) $$w=\int_{0}^{\varepsilon }\sigma \left(\varepsilon \right)d\varepsilon$$

where *σ*(*ε*) corresponds to the stress associated with strain, expressed in MPa. Plateau stress and densification strain are the key characteristics related to the properties of the energy absorption of porous structures. The principal energy absorption stage of the structure occurs during the plateau stage. The effective compression stroke for energy absorption extends from the initiation of external loading to the point where the structure is compressed into a dense state. Moreover, the moment at which the structure transitions into this dense state is defined by Equation (4).

(4) $${\left.\frac{d\eta \left(\varepsilon \right)}{d\varepsilon }\right|}_{\varepsilon =\varepsilon_{e}}=0$$

where *η*(*ε*) denotes the efficiency of energy absorption of the structure, which is a function of structural strain. Equation (4) reveals that the structure begins to enter a densified state when the efficiency of energy absorption reaches its maximum, at which point the strain of the structure is at the initial densification strain $\varepsilon_{e}$. The equation of energy absorption efficiency is depicted in Equation (5).

(5) $$\eta \left(\varepsilon \right)=\frac{w}{\sigma \left(\varepsilon \right)}$$

Using Equation (5), the stress–strain curve of the structure can be converted into a curve of efficiency of energy absorption, as depicted in Figure 6. The area shaded in gray, bounded by the stress–strain curve and the curve of efficiency of energy absorption, represents the total energy absorbed by the structure during its compression stroke.

## 3. Results and Discussion

### 3.1. Analysis of Experimental Results

**Figure 7:**
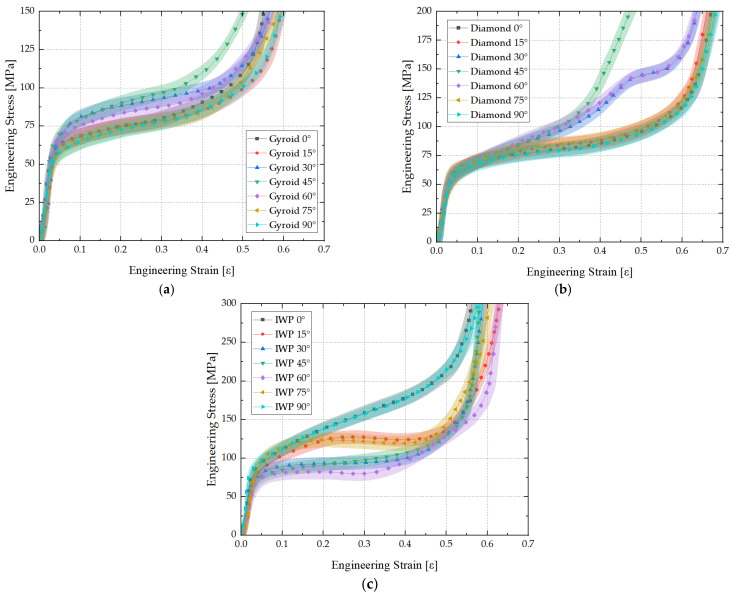
Stress–strain curves with different load-bearing angles: (**a**) Gyroid, (**b**) Diamond, (**c**) IWP.

Quasi-static compression tests were conducted on Gyroid, Diamond, and IWP structures under load-bearing angles of 0°, 15°, 30°, 45°, 60°, 75°, and 90°. Each structure underwent six repeated experiments, and the resulting six sets of experimental data were subjected to error analysis. The mean and standard deviations of the six sets of data were calculated. Using the mean and standard deviations, error bands were plotted, as shown in Figure 7. The small discrepancies between the six sets of experiments and the high degree of data congruence indicate the reliability of the experimental results.

The curves revealed that altering the load-bearing angle of Gyroid structures does not result in a significant variation in their stress–strain relationship, as shown in Figure 7a. Moreover, at 30°, 45°, and 60°, the plateau stage of the structural stress–strain curve was slightly higher compared to other angles, indicating enhanced load-bearing capacity at these orientations. In contrast, the characteristics of Diamond and IWP structures exhibited greater sensitivity to changes in the load-bearing angle, as shown in Figure 7b,c. The stress plateau stage of the Diamond structure was notably shortened at 45°, leading to early densification. At 30° and 60°, the stress–strain curve of the Diamond structure demonstrated graded energy absorption characteristics, prolonging the duration of the stress plateau and increasing plateau stress, thereby showcasing the excellent performance of energy absorption. As for the IWP structure, a slight decrease in internal stress was observed at 15° and 75°. Furthermore, apart from 0° and 90°, the stress–strain curve of the IWP structure at other angles resulted in prolonged durations of stress plateau, yet with a substantial decrease in plateau stress, thereby compromising the capability of energy absorption of the structure.

**Figure 8:**
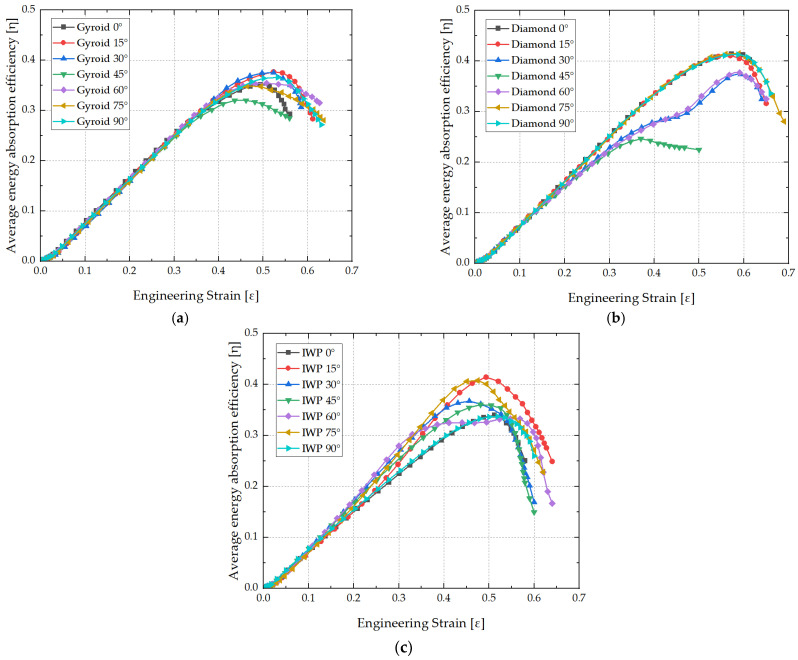
Average energy absorption efficiency curves with different load-bearing angles: (**a**) Gyroid, (**b**) Diamond, (**c**) IWP.

Efficiency of energy absorption is regarded as a critical metric for assessing the performance of the energy absorption of structures. A high efficiency of energy absorption indicates that the structure can effectively dissipate the energy stored in internal stresses. Using Equations (3) and (5), the stress–strain curves were further transformed into the curves of efficiency of energy absorption, as shown in Figure 8. It was observed that as the compression process of energy absorption of the TPMS structures continues, the efficiency of energy absorption gradually increases until it reaches a peak at the densification point. Subsequently, the characteristics of TPMS structures gradually become denser, and their energy absorption efficiency rapidly decreases. At this point, it indicated that TPMS structures no longer absorbs additional energy but rather transfers it to the other side through their dense structures. Notably, for Gyroid and Diamond structures, the maximum efficiency of energy absorption significantly decreases at 45°, which is closely related to the shortened stress plateau stage (early densification). Additionally, the maximum efficiency of energy absorption of the Diamond structure at 30° and 60° were also significantly reduced. At 15° and 75°, the maximum efficiency of energy absorption of the IWP structure was the highest at all angles. Based on the preceding analysis, it is evident that the sudden increase in internal stress during the stress plateau stage can lead to a decrease in the maximum efficiency of energy absorption. Such an abrupt increase in internal stress may trigger secondary or even multiple plateaus of energy absorption. Conversely, a sudden decrease in stress during the plateau process can enhance the maximum efficiency of energy absorption. This phenomenon occurred because the continuous compression energy absorption led to a secondary rise in internal stress, thereby improving the maximum efficiency of energy absorption.

**Figure 9:**
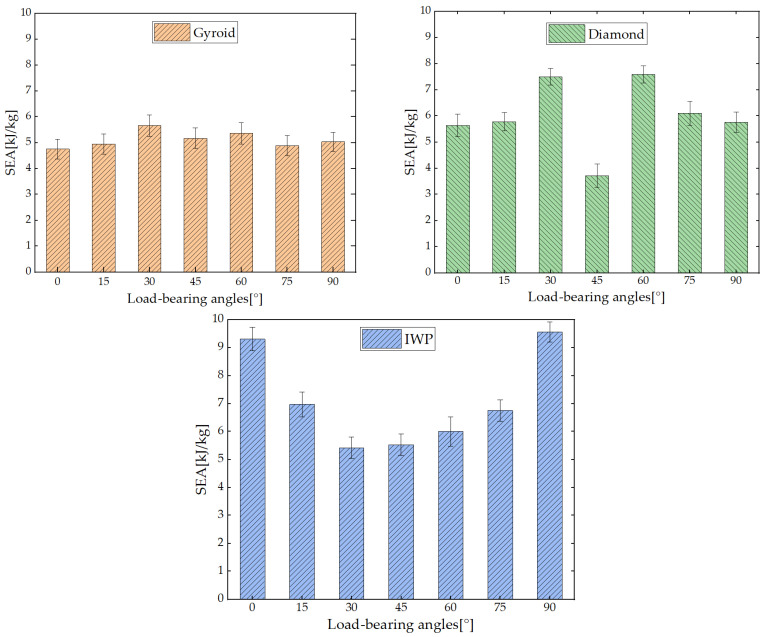
SEA of Gyroid, Diamond and IWP structures at different load-bearing angles.

In this study, 316L steel was chosen as the solid material for TPMS structures, with a porosity of 70% and a mass per cubic meter of 7980 kg. The total energy absorption and mass of the structure were calculated, and the SEA of Gyroid, Diamond, and IWP structures was determined using Equation (2). Figure 9 illustrates the SEA of the three TPMS structures.

The results from Figure 9 demonstrated that at 0°, 15°, 75°, and 90°, the SEA of the Gyroid structure remained relatively constant, with only a certain degree of enhancement observed at 30°, 45°, and 60°. In contrast, the SEA of the Diamond structure exhibited sensitivity to the load-bearing angle. Particularly at 30° and 60°, the capability of energy absorption of the Diamond structure significantly increased, with ratios of energy absorption approaching 8 kJ/kg, while at 0°, 15°, 75°, and 90°, it remained around 6 kJ/kg. Additionally, at 45°, the SEA of the Diamond structure experienced a sharp decrease, dropping to values below 4 kJ/kg. The characteristics of energy absorption of the IWP structure differed from the preceding two. While maintaining a high level of energy absorption at 0° and 90°, the capability of energy absorption at other angles exhibited a notable decline. Specifically, at 30°, 45°, and 60°, it decreased to 6 kJ/kg or below, while at 15° and 75°, it diminished to around 7 kJ/kg. Additionally, although altering the load-bearing angles of the three TPMS structures resulted in varying degrees of changes in their performance of energy absorption, all three structures exhibited a symmetric trend centered around 45°. To more deeply explore the analysis of the differences in the performance of the energy absorption of the three TPMS structures at various load-bearing angles, Table 4, Table 5 and Table 6 present the densification strain and plateau stress of these structures.

Table 4 presents a comparative analysis of the performance of energy absorption of the Gyroid structure at different load-bearing angles. The data indicate that the plateau stresses at load-bearing angles of 0°, 15°, 75°, and 90° generally stabilized around 80 MPa, while there were slight variations during densification, albeit minimal. Thus, the differences in SEA for the Gyroid structure at these four angles were less than 5%, which can be considered practically equivalent in applications in engineering fields. Although the densification strains at 30° and 60° were also approximately 0.5, their plateau stresses significantly increased, resulting in respective increases of 19% and 14% in SEA compared to other angles. Furthermore, at 45°, the densification strain was approximately 0.45, approximately 5% higher than at other angles. However, due to the plateau stress reaching the highest level among all angles, namely 95 MPa, the SEA of structures at 45° still increased by 10%. A comparison of the energy absorption performance of the Diamond structure was presented at different load-bearing angles, as shown in Table 5. Similar to Gyroid structure, the variation in SEA was not pronounced at 0°, 15°, 75°, and 90°. However, at 30° and 60°, there was a respective increase of 33% and 34% in SEA, with plateau stresses reaching 105 MPa. This was attributed to the Diamond structure exhibiting a secondary plateau of energy absorption, enhancing their internal loading capacity. Conversely, at 45°, there was no significant change in plateau stress, but densification occurred approximately 20% earlier. Premature densification led to a shortened duration of the plateau stage, resulting in a 34% decrease in SEA. According to the data in Table 6, for the IWP structure, the SEA was significantly higher at 0° and 90° compared to other angles. In contrast, at 15°, 30°, 45°, 60°, and 75°, the SEA decreased by 25%, 42%, 40%, 36%, and 24%, respectively. Meanwhile, the plateau stresses at 0° and 90° exceeded 150 MPa, which was the highest among all angles. Plateau stresses at 15° and 75° were approximately 120 Mpa, while at 30°, 45°, and 60°, the stresses dropped to 95 Mpa.

From the comprehensive comparison of the three structures above, it was evident that while the Gyroid structure exhibited minimal variation across different load-bearing angles, its capacity for energy absorption was the lowest among the three structures. Conversely, significant differences were demonstrated in the Diamond and IWP structures in energy absorption at various load-bearing angles, yet they could achieve superior performance of energy absorption at specific angles. This indicates that different structures have their respective advantages in energy absorption.

### 3.2. Analysis of Deformation Behavior

To explore the mechanisms underlying the impact of load-bearing angles on the performance of the energy absorption of structures, this section extensively discusses the deformation and damage experienced by the structure during the process of compression. By elucidating the internal changes and damage mechanisms within the structure, this study analyzed the fundamental factors contributing to variations in the capacity of energy absorption.

During the processes of quasi-static compression, structures primarily absorb external energy through plastic deformation. Typically, TPMS structures exhibit two predominant deformation modes under compression loading: tension-dominated and bending-dominated modes [44]. In structures where bending deformation prevails, compression leads to rapid entry into the stress plateau stage after overall yielding, primarily due to plastic collapse at the edges of the unit cells [45]. Conversely, in structures dominated by tension deformation, compression induces evident post-yield softening as a result of brittle collapse or the plastic buckling of supporting struts. Following the initial post-yield softening, the stress value of the structure remains nearly constant as strain increases. After experiencing the stress plateau stage, regardless of the deformation mode, the structure gradually densifies due to increased loading capacity. Furthermore, for structures with the same porosity, the stress–strain curves of tension-dominated and bending-dominated structures can be compared. Tension-dominated structures typically exhibit higher strength and stiffness, while bending-dominated structures demonstrate more prolonged stress plateau regions [46,47].

**Figure 10:**
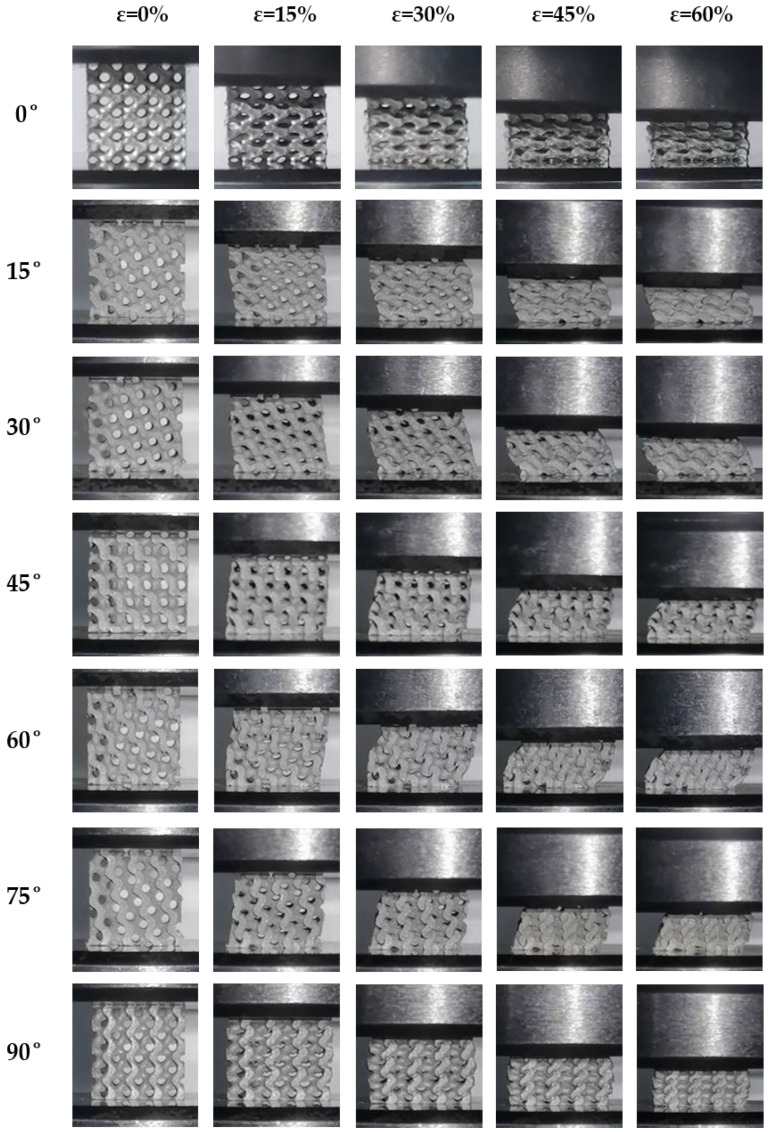
Deformation of the Gyroid structure with different loading angles.

The deformation of the Gyroid structure was observed during the process of compression, as shown in Figure 10. At 0° and 90°, the compression of the Gyroid structure primarily induced axial deformation, accompanied by distortion in the thin-walled regions of the structure. These areas were relatively weaker and more susceptible to localized deformation, thereby confirming that the deformation of the Gyroid structure was bending-dominated during compression [14,48]. Throughout the process of compression, cell connections gradually twisted and squeezed together, leading to the progressive collapse of the structure, indicating its transition to a densified state where pore spaces at nodal connections were compacted, aligning with the strain-hardening effect observed in other literature [49]. At 15° and 75°, slight horizontal displacements occurred during compression, along with plastic collapse at the edges of the units. As compression continued, the overall structure experienced squeezing and gradually entered a densified state, with this phenomenon becoming more pronounced at larger inclination angles. At 30° and 60°, the horizontal displacement deformation of the structure was most significant, with more evident local plastic collapse. Initially, localized yielding collapse occurred at the edges of the structure, followed by a spreading collapse throughout the entire structure. Combined with the earlier observation of a slight increase in the plateau stress at 30° and 60°, it was evidenced that the phenomenon of localized collapse yielding can concentrate and enhance internal stresses within the structure to some extent. Overall, when subjected to external compressive loads, the deformation of the Gyroid structure was primarily bending at nodal connections. Its yielding behavior manifested as localized yielding failures at node connections, without the presence of distinct yielding bands being observed.

**Figure 11:**
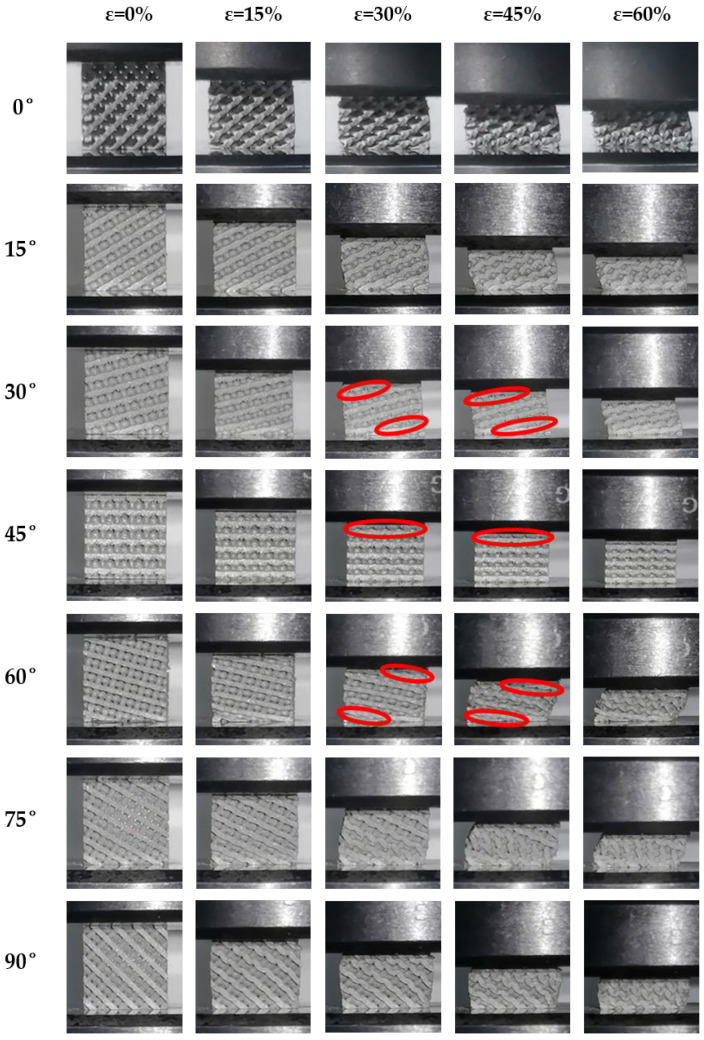
Deformation of the Diamond structure with different loading angles (red circle: yield belt).

The deformation process of the Diamond structure under compressive loads is shown in Figure 11. These images reveal that at 0°, 45°, and 90°, the significant lateral displacement of the Diamond structure was not manifested. Notably, at 45°, the deformation in the vertical direction (Z-axis direction) was extremely small, almost immediately transitioning into a densified state. This indicated a significant strain-hardening phenomenon, leading to a notable reduction in the stress plateau, resulting in a decrease in the SEA by over 30%. At 15° and 75°, the deformation of the Diamond structure was slight lateral shear, with relatively uniform stress distribution throughout the deformation process, and no evident shear bands or localized squeezing deformation were observed. However, at 30° and 60°, significant lateral displacement was manifested, accompanied by layer-by-layer squeezing, with one side experiencing squeezing deformation and the other side undergoing tensile deformation. As compression progressed, at 30° and 60°, the structure was progressively collapsed layer by layer under external loads until the final layer was squeezed, leading to an increase in internal stress in the overall structure, forming a secondary plateau. After a period of the secondary plateau effect, the overall structure began to transition into a densified state. For the Diamond structure, while significantly enhancing SEA at 30° and 60°, the performance was somewhat weakened at 45°, indicating that the Diamond structure was more sensitive to load-bearing angles compared to the Gyroid structure. In contrast, the energy absorption performance of the Gyroid structure was relatively stable, but its energy absorption capacity was low, making it unsuitable for high-energy absorption scenarios.

**Figure 12:**
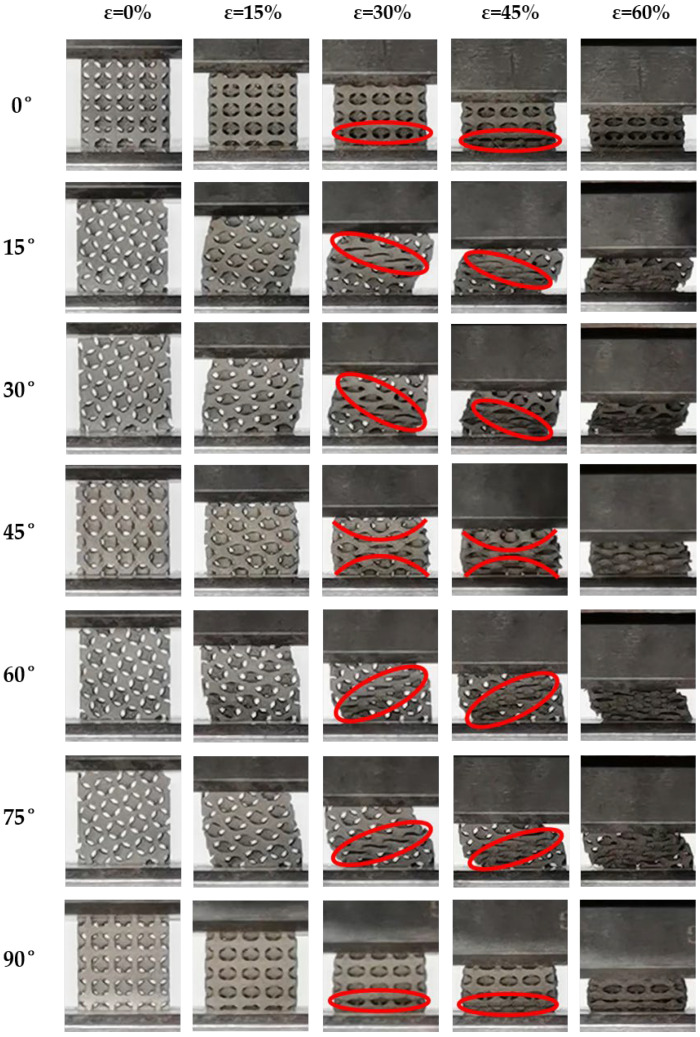
Deformation of the IWP structure with different loading angles (red circle: yield belt).

More pronounced characteristics compared to the other two TPMS structures were exhibited for IWP structure, as shown in Figure 12. Apart from 0° and 90°, the performance of the energy absorption of the IWP structure was weakened to varying degrees at other angles. The IWP structure was supported by struts, and at 0° and 90°, its load-bearing struts were parallel to the loading direction, thereby maximizing resistance to deformation. Conversely, at other angles, the struts of the IWP structure were inclined at certain angles to the loading direction, resulting in buckling deformation (compression on one side and tension on the other), leading to deformation and displacement in the shear direction of the structure. At 15° and 75°, where the angle between the struts of the IWP structure and the loading direction was smaller, buckling accompanied by the emergence of local shear bands began as compression occurred. As the angle between the struts of the IWP structure and the loading direction increased, the degree of buckling intensified, and the shear bands propagated to the diagonal position of the entire structure. At 45°, an X-shaped shear band appeared in the structure under compression loading, where the struts of the IWP structure adopted a cross-sectional load-bearing configuration. However, the collapse of the structure was rapid due to the insufficient capacity of axial load-bearing, without demonstrating significant deformation resistance. Additionally, the local collapse yielding prior to the overall structure was exhibited under all loading angles. Following the yielding softening induced by local collapse, the stress plateau of the structure remained relatively constant, as evidenced by the stress–strain curves of the IWP structure presented earlier. Therefore, under compressive loading, the typical characteristics of tensile deformation were exhibited within the IWP structure.

It should be noted that, as the experiment progressed, slight angles between the upper and lower compression platens were observed, which was caused by the failures of the structures. However, these angles were very small and were disregarded in this study. Further research can delve deeper into this limitation.

In summary, altering the load-bearing angles of the three TPMS structures resulted in distinct variations in their performance in terms of energy absorption and deformation characteristics. As for the Gyroid structure, its performance in terms of energy absorption demonstrated a highly stable response to changes in load-bearing angle but exhibited a lower level of energy absorption, primarily characterized by bending deformation during compression. During compression, the deformation of the Diamond structure was predominantly bending, but at 30°, 45°, and 60°, it also exhibited some characteristics of tensile deformation. At 45°, it tended to prematurely enter a densification stage, shortening the stress plateau and consequently reducing energy absorption. However, at 30° and 60°, the formation of secondary plateaus of energy absorption significantly enhanced its capacity for energy absorption. In contrast, during compression, the typical characteristics of tensile deformation were exhibited within the IWP structure, rendering it highly sensitive to changes in load-bearing angles. Under certain conditions, its capacity for axial load-bearing decreased significantly, resulting in a severe reduction in the capability of energy absorption.

## 4. Conclusions

In this study, Gyroid, Diamond, and IWP structures made from 316L steel were fabricated using SLM technology. Quasi-static compression tests were conducted on these structures to analyze the variations in the capabilities of energy absorption at different loading angles. The deformation and failure modes during the compression process were also analyzed to elucidate the reasons behind these changes. The main conclusions are as follows.

- Adjusting the load-bearing angles of the Gyroid, Diamond, and IWP structures resulted in distinct responses of energy absorption under quasi-static compressive loads. The performance of energy absorption of the Gyroid structure remained the most stable when changing the load-bearing angle, maintaining nearly consistent capability for energy absorption at all angles. For the Diamond structure, similar performances of energy absorption were observed at 0°, 15°, 75°, and 90°. However, there was a significant enhancement in SEA at 30° and 60°, while a notable reduction occurred at 45°. In contrast, the performance in energy absorption of the IWP structure was most sensitive to changes in the load-bearing angle. It maintained good performance in energy absorption only at 0° and 90°, with a substantial decrease in the capabilities for energy absorption at other angles. Additionally, the SEA of all three structures exhibited a symmetrical pattern centered at 45°.
- Under compressive loading, the deformations of the Gyroid structure were primarily characterized by bending. Similarly, the behaviors of bending-dominated deformation were predominantly shown by the Diamond structure at 0°, 15°, 75°, and 90°. However, at 30° and 60°, a mixed mode of bending and tensile deformations was displayed within the Diamond structure, thereby introducing a secondary plateau of energy absorption that effectively increased the plateau stress of the structure. At 45°, significant strain hardening was demonstrated. As for the IWP structure, its deformation exhibited typical characteristics of tensile deformation under compressive loads. Apart from maintaining good capabilities for load-bearing at 0° and 90° through its structural struts, evident failures of local buckling were shown at other loading angles, which weakened the capacity of overall load-bearing, thereby reducing its plateau stress and capabilities of energy absorption.
- When the deformation mode of the TPMS structures was primarily tensile, a stronger load-bearing capacity was exhibited. Consequently, a higher capacity of specific energy absorption was exhibited. In contrast, compared to structures primarily deforming through tensile, higher stability under multidirectional loads can be shown in those primarily deforming through bending. In summary, although structures that rely on bending deformation may not perform as well as those that rely on tensile deformation on energy absorption, they can provide better stability. Additionally, structures primarily deforming through tensile deformations may have poorer stability, but their potential for energy absorption was higher.

## Figures and Tables

**Table 1:** Chemical composition of 316L steel.

Element	Fe	Cr	Ni	Mo	C	Other
**Weight percent (%)**	Balance	18	12	2	<0.03	<1.0

**Table 2:** The parameters of 316L steel.

Young’s Modulus	Density	Poisson Ratio	Yielding Stress
176 GPa	7980 kg/m^3^	0.3	480 MPa

**Table 3:** Parameters of the SLM manufacturing process.

Laser Power	Layer Thickness	Scan Speed	Printing Environment
140 W	30 μm	650 mm/s	Argon

**Table 4:** Comparison of energy absorption performance of the Gyroid structure with different angles.

Load-Bearing Angle	SEA (kJ/kg)	Densification Strain	Plateau Stress (MPa)
0°	4.75 ± 0.38	0.504 ± 0.036	80.34 ± 4.13
15°	4.95 ± 0.39	0.524 ± 0.033	80.21 ± 3.89
30°	5.66 ± 0.41	0.525 ± 0.035	93.75 ± 3.56
45°	5.17 ± 0.40	0.461 ± 0.040	95.01 ± 4.07
60°	5.36 ± 0.42	0.507 ± 0.038	88.95 ± 4.23
75°	4.89 ± 0.39	0.493 ± 0.036	80.24 ± 3.93
90°	4.92 ± 0.37	0.533 ± 0.037	79.96 ± 4.42

Note: The data = average ± standard deviations.

**Table 5:** Comparison of energy absorption performance of the Diamond structure with different angles.

Load-Bearing Angle	SEA (kJ/kg)	Densification Strain	Plateau Stress (MPa)
0°	5.64 ± 0.43	0.572 ± 0.032	82.49 ± 4.15
15°	5.78 ± 0.35	0.570 ± 0.033	84.34 ± 4.23
30°	7.50 ± 0.32	0.594 ± 0.035	105.95 ± 5.06
45°	3.72 ± 0.45	0.370 ± 0.039	85.77 ± 4.57
60°	7.59 ± 0.33	0.591 ± 0.032	107.12 ± 4.55
75°	6.10 ± 0.46	0.589 ± 0.038	86.16 ± 4.98
90°	5.76 ± 0.39	0.587 ± 0.041	81.74 ± 4.24

Note: The data = average ± standard deviations.

**Table 6:** Comparison of energy absorption performance of the IWP structure with different angles.

Load-Bearing Angle	SEA (kJ/kg)	Densification Strain	Plateau Stress (MPa)
0°	9.30 ± 0.42	0.532 ± 0.037	152.88 ± 4.37
15°	6.97 ± 0.44	0.499 ± 0.041	119.16 ± 4.18
30°	5.42 ± 0.39	0.496 ± 0.036	93.97 ± 4.86
45°	5.53 ± 0.38	0.498 ± 0.039	96.60 ± 4.54
60°	6.04 ± 0.52	0.499 ± 0.044	95.86 ± 5.02
75°	6.75 ± 0.38	0.492 ± 0.045	120.41 ± 4.73
90°	9.55 ± 0.36	0.538 ± 0.032	153.97 ± 5.14

Note: The data = average ± standard deviations.

## Data Availability

Data are contained within the article.
